# *Clostridioides difficile* infection in Saudi Arabia: Prevalence, risk factors, and outcomes in a tertiary hospital setting

**DOI:** 10.1371/journal.pone.0340075

**Published:** 2025-12-31

**Authors:** Abdullah M. Aldhaif, Mohammed A. Al-Garni, Ahmed A. Muyidi, Mohammed H. Makkawi

**Affiliations:** 1 Molecular and Medical Microbiology Laboratory, King Abdulaziz University Hospital, Jeddah, Saudi Arabia; 2 Molecular and Medical Microbiology Laboratory, King Faisal Medical City, Abha, Saudi Arabia; 3 Medical Laboratory Science, Applied Science Medical College, King Khalid University, Saudi Arabia; Texas A&M University College Station: Texas A&M University, UNITED STATES OF AMERICA

## Abstract

**Background:**

*Clostridioides difficile* infection (CDI) remains a major healthcare-associated infection with limited contemporary data from Saudi Arabia. This study evaluated CDI prevalence, risk factors, recurrence predictors, and treatment patterns in hospitalized patients at a tertiary teaching hospital.

**Methods:**

Retrospective analysis of 1,054 hospitalized patients screened between March 2023 and February 2024. CDI was confirmed by positive toxin assay and/or nucleic acid amplification test (NAAT). Demographic, clinical, antibiotic, acid-suppressant, and treatment data were collected. Bivariate associations and multivariable logistic regression were used to identify predictors of recurrence.

**Results:**

CDI prevalence was 10.8% (114/1,054). Median age was 37 years (IQR 51.25); 32.5% had malignancy. Antibiotic exposure preceded CDI in 59.6% (meropenem 24.7%, ceftriaxone 16.5% of courses), and acid suppressants were used in 57.0% (omeprazole 92.3%). Recurrence occurred in 11.4% (13/114). On bivariate analysis, acid-suppressant use was significantly associated with recurrence (*p* = 0.041). In multivariable logistic regression, only metronidazole plus vancomycin combination therapy independently predicted recurrence (OR 11.29, 95% CI 1.13–112.42, *p* = 0.039). Trends were observed for malignancy (OR 2.94, p = 0.112) and acid-suppressant use (OR 1.85, p = 0.440), limited by the small number of recurrent events. Metronidazole monotherapy dominated treatment (64.8%).

**Conclusion:**

CDI prevalence reached 10.8% with an 11.4% recurrence rate. Acid-suppressant exposure and combination therapy were key recurrence signals, while metronidazole remains overused despite international guideline shifts. Enhanced antibiotic and acid-suppressant stewardship, alongside improved access to guideline-recommended therapies, are critical to reducing CDI burden in Saudi tertiary hospitals.

## 1. Background

*Clostridioides difficile* is a gram-positive, spore-forming anaerobic bacterium that causes gastrointestinal infections, ranging from mild diarrhea to life-threatening colitis [1,2]. *Clostridioides difficile* infection (CDI) has emerged as a significant healthcare concern, with increasing incidence rates observed in recent years [3–5]. CDI is primarily mediated by two toxins, TcdA and TcdB, which disrupt host cell function, leading to fluid loss and diarrhea [6]. Spores are crucial in CDI transmission, persistence, and recurrence [7,8]. Diagnosis typically involves detecting *C. difficile* toxins in feces [1]. Current treatment guidelines recommend vancomycin or fidaxomicin as first-line therapies, with metronidazole reserved for mild cases in younger patients [9,10]. For recurrent CDI, bezlotoxumab may be used as an adjunctive therapy [9,10]. Fecal microbiota transplantation is highly effective for multiple recurrences, with success rates exceeding 85% [10]. Prevention strategies include proper hygiene, judicious antibiotic use, and early detection [11]. Prevention measures focus on hand hygiene, contact precautions, and environmental cleaning to reduce transmission [12].

A 2008 survey funded by the European Center for Disease Prevention and Control (ECDC) reported a 71% increase in CDI incidence compared to previous European surveillance studies [13]. Consequently, the Centers for Disease Control and Prevention (CDC) and other institutions have undertaken significant efforts to prevent infections and implement epidemiological surveillance, leading to a reduction in the standardized infection ratio and the overall disease burden of CDI [14]. However, the incidence and severity of *CDI* have increased significantly in recent years, affecting both pediatric and adult populations [2,12]. Studies across different countries report similar epidemiological trends, with CDI predominantly affecting older patients, particularly those over 65 years [3,15]. Healthcare-associated infections are prevalent, but community-associated cases are also significant [3,15]. The main risk factor for healthcare-associated CDI is the inappropriate, prolonged, and cumulative use of antibiotics like clindamycin, penicillin, quinolones, and carbapenems [16]. These antibiotics disrupt the intestinal microbiota, promoting the overgrowth of *C. difficile*, which increases the risk of infection for up to three months after stopping the medication [17]. Other risk factors that increase the likelihood of CDI include being over 65 years old, recent hospitalization or extended stays in healthcare facilities, recent gastrointestinal surgery, and the use of certain medications like proton pump inhibitors (PPIs), non-steroidal anti-inflammatory drugs (NSAIDs), and immunosuppressants. The use of acid-suppressing agents, in particular, elevates the risk of CDI by disrupting the natural gut microbiota [18,19]. CDI recurrence rates range from 16.7% to 22% [4,3]. Mortality rates are disproportionately high among older patients, with deaths occurring shortly after diagnosis [4,15]. Seasonal variations in CDI incidence have been observed, with higher rates in winter and spring [5].

CDI incidence in Saudi Arabian hospitals appears relatively low compared to high-income countries. Studies report rates ranging from 0.9–3.5 per 10,000 patient days [20–22]. The highest incidence was observed in intensive care units and immunocompromised patient wards [22]. Common risk factors include acid-reducing drugs, prolonged antibiotic use, and extended hospitalization [21,22]. However, some patients developed CDI without prior antibiotic exposure [20]. Most cases were healthcare-onset, with a smaller proportion being community-onset [20–22]. Compliance with treatment guidelines (IDSA/SHEA, ACG, ESCMID) was associated with improved clinical outcomes, including higher cure rates and lower mortality.

Despite increasing reports from the Middle East, contemporary data on CDI in Saudi Arabia remain limited, particularly regarding recurrence predictors and the impact of non-antibiotic risk factors such as proton pump inhibitors. This retrospective study at a tertiary teaching hospital in Saudi Arabia aimed to determine CDI prevalence among hospitalized patients, characterize antibiotic and acid-suppressant exposure patterns, identify clinical factors associated with recurrence, and evaluate predictors of recurrent CDI using multivariable logistic regression.

## 2. Methods

### 2.1. Study design and setting

This retrospective study was conducted at King Abdulaziz University Hospital (KAUH) in Saudi Arabia. The objective was to determine the prevalence, incidence rates, risk factors, and outcomes associated with CDI among hospitalized patients.

### 2.2. Data collection

#### 2.2.1. Study population.

The study included all hospitalized patients at KAUH from March 2023 to February 2024. Patients were selected based on the presence of CDI, determined by clinical records and laboratory test results.

#### 2.2.2. Inclusion criteria.

Patients, regardless of their age, with a confirmed diagnosis of CDI based on positive laboratory tests for *C. difficile* toxins in feces, including toxin protein, glutamate dehydrogenase antigen (GDH), and/or *C. difficile* toxin nucleic acid amplification test (NAAT).

#### 2.2.3. Exclusion criteria.

Patients with incomplete medical records. Patients who were not hospitalized during the study period.

### 2.3. Data sources

Data were extracted from the hospital’s electronic health records (EHR) system. Information collected included patient demographics, clinical history, antibiotic use, underlying health conditions, length of hospital stay, CDI diagnosis details, treatment regimens, and outcomes.

### 2.4. Data collection methods

In the data collection process for this study, CDI cases were identified through diagnostic codes and laboratory reports confirming positive tests for *C. difficile* toxins in feces. To ensure accurate diagnosis and gather comprehensive clinical data, medical records were meticulously reviewed. The data variables collected encompassed several categories. Demographic information included patient age, sex, and comorbidities. Clinical data involved details such as hospital admission records, length of stay, prior antibiotic use, and the use of acid-suppressing agents. Risk factors considered included recent gastrointestinal surgeries, use of immunosuppressants, and other potential CDI risk factors. Finally, treatment and outcomes data were gathered, including the type of treatment administered, the patient’s response to this treatment, instances of CDI recurrence, and mortality rates.

### 2.5. Statistical analysis

Descriptive statistics were used to summarize patient demographics, clinical characteristics, and treatment variables. Continuous variables, such as age and duration of antibiotic use, were presented as medians with interquartile ranges (IQR), while categorical variables were summarized as frequencies and percentages.

The prevalence of CDI was determined as the proportion of confirmed CDI cases relative to the total tested population. Cross tabulations were performed to examine associations between CDI recurrence (R-CDI) and categorical predictors, including demographic, clinical, and therapeutic factors (e.g., malignancy, previous surgery, antibiotic exposure, and acid-suppressant use).

The Chi-square test or Fisher’s Exact test (when expected cell counts were <5) was used to assess statistical associations between variables and recurrence; A multivariable logistic regression model was then constructed to identify independent predictors of recurrence. Variables were selected based on their clinical relevance in prior literature, biological plausibility, and treatment-related importance (e.g., malignancy, acid-suppressant use, and specific CDI therapies). Ward category, nationality, and detailed medical history variables were not included in the model due to weak or non-significant associations with recurrence in the bivariate analysis, and to avoid overfitting given the limited number of recurrence events. Model performance was evaluated using −2 log-likelihood, Nagelkerke R^2^, and the Hosmer–Lemeshow goodness-of-fit test. Adjusted odds ratios (ORs) with 95% confidence intervals (CIs) were reported. All analyses were performed using IBM SPSS Statistics version 25 (IBM Corp., Armonk, NY, USA).

### 2.6. Ethical approval

All methods were performed in accordance with relevant institutional guidelines and regulations. This retrospective study was approved by the Unit of Biomedical Ethics Research Ethics Committee (REC) at King Abdulaziz University Hospital, Jeddah, Saudi Arabia (Reference number 527−23). The need for informed consent was waived by the REC, as the study was a non-interventional retrospective record review.

The data were accessed for research purposes on March 15, 2024. During data collection, the authors had access to identifiable patient information, which was used solely for accurate clinical matching and data verification. All personal identifiers were removed prior to analysis to ensure confidentiality and data privacy.

## 3. Result

### 3.1. Study population and baseline characteristics

Demographic and clinical characteristics were evaluated in 114 patients with CDI, with stratification by recurrence status to identify potential risk profiles. The data sequentially by demographics, admission ward, and comorbidities to establish a comprehensive patient profile. Among 1,054 hospitalized patients screened, the prevalence of CDI was 10.8% (n = 114). The median age was 37.0 years (IQR 51.25), comprising 27.2% (n = 31) aged <18 years, 36.8% (n = 42) aged 18–60 years, and 36.0% (n = 41) aged >60 years. Males represented 51.8% (n = 59) of cases, and non-Saudi nationals accounted for 55.3% (n = 63). Patients were admitted to various wards, predominantly emergency (21.9%, n = 25), pediatrics (15.8%, n = 18), and surgery (15.8%, n = 18). Prevalent comorbidities included malignancy in 32.5% (n = 37) and gastrointestinal disease in 20.2% (n = 23), whereas 13.2% (n = 15) had no recorded prior diagnosis. Non-recurrent CDI occurred in 101 patients (88.6%), and recurrent CDI (R-CDI) in 13 (11.4%), with greater proportions of older age and malignancy observed in the R-CDI group. These findings indicate a diverse patient population burdened by underlying conditions (Table 1).

**Table 1:** Baseline demographic and clinical characteristics of hospitalized patients with CDI (N = 114).

Characteristic	Category	Total (n = 114)	CDI without recurrence (n = 101)	Recurrent CDI (R-CDI) (n = 13)
**Age group (years)**	< 18 years	31 (27.2%)	28 (27.7%)	3 (23.1%)
	18–60 years	42 (36.8%)	40 (39.6%)	2 (15.4%)
	> 60 years	41 (36.0%)	33 (32.7%)	8 (61.5%)
**Sex**	Male	59 (51.8%)	53 (52.5%)	6 (46.2%)
	Female	55 (48.2%)	48 (47.5%)	7 (53.8%)
**Nationality**	Saudi	51 (44.7%)	45 (44.6%)	6 (46.2%)
	Non-Saudi	63 (55.3%)	56 (55.4%)	7 (53.8%)
**Hospital ward**	Emergency	25 (21.9%)	20 (19.8%)	5 (38.5%)
	Pediatrics	18 (15.8%)	15 (14.9%)	3 (23.1%)
	Gastroenterology	15 (13.2%)	14 (13.9%)	1 (7.7%)
	Surgery	18 (15.8%)	16 (15.8%)	2 (15.4%)
	Medical	12 (10.5%)	10 (9.9%)	2 (15.4%)
	ICU	11 (9.6%)	11 (10.9%)	0 (0.0%)
	Specialized clinics	9 (7.9%)	9 (8.9%)	0 (0.0%)
	Hematology & Oncology	6 (5.3%)	6 (5.9%)	0 (0.0%)
**Comorbidity category**	Malignant neoplasm	37 (32.5%)	30 (29.7%)	7 (53.8%)
	Gastrointestinal disease	23 (20.2%)	20 (19.8%)	3 (23.1%)
	No diagnosis	15 (13.2%)	14 (13.9%)	1 (7.7%)
	Cardiovascular disease	12 (10.5%)	11 (10.9%)	1 (7.7%)
	Blood/ immune disorder	4 (3.5%)	4 (4.0%)	0 (0.0%)
	Miscellaneous conditions	7 (6.1%)	7 (6.9%)	0 (0.0%)
	Infectious diseases	6 (5.3%)	6 (5.9%)	0 (0.0%)
	Endocrine/ metabolic disorder	4 (3.5%)	3 (3.0%)	1 (7.7%)
	Renal/ urological disorder	4 (3.5%)	4 (4.0%)	0 (0.0%)
	Neurological disorder	2 (1.8%)	2 (2.0%)	0 (0.0%)
**Age, Median (IQR)**		37.0 (51.25)	36.0 (51.0)	65.0 (46.0)

**Note:** Percentages are calculated out of the total study (N = 114). Values may not sum to 100% due to rounding.

**Abbreviation:** IQR – interquartile range; ICU – intensive care unit.

### 3.2. Antibiotic exposure

Antibiotic exposure preceding CDI onset was assessed as a principal risk factor, with results stratified by recurrence status for comparative analysis. The analysis was structured to progress from exposure prevalence and number of agents to specific antibiotics, administration route, and duration, reflecting clinical prescribing sequences. Antibiotics were administered to 59.6% (n = 68) of patients, with no exposure or missing data in 40.4% (n = 46). Among those exposed, 79.4% (n = 54) received one antibiotic type, 16.2% (n = 11) two types, and 4.4% (n = 3) three types. A total of 85 antibiotic courses were documented across these 68 patients, with a median duration of 6.75 days (IQR 7.0) and concurrent or sequential combinations in 20.6% (n = 14). The most common agents were meropenem (24.7% of courses, n = 21), ceftriaxone (16.5%, n = 14), piperacillin–tazobactam (9.4%, n = 8), and ciprofloxacin (9.4%, n = 8). Intravenous administration predominated (77.6%, n = 66) over oral (22.4%, n = 19). Patterns were largely similar between groups, although meropenem use was higher in R-CDI (37.5% of courses). Broad-spectrum intravenous antibiotics were frequently employed, which may contribute to CDI development but showed no evident association with recurrence (Table 2).

**Table 2:** Antibiotic exposure patterns and duration among hospitalized patients with CDI (N = 114).

Characteristic	Category	Total (n = 114)	CDI without recurrence (n = 101)	Recurrent CDI (R-CDI) (n = 13)
Antibiotic exposure	No antibiotic/ No data	46 (40.4%)	40 (39.6%)	6 (46.2%)
	Used antibiotics (≥1 type)	68 (59.6%)	61 (60.4%)	7 (53.8%)
Number of antibiotic types (a)	One	54 (79.4%)	48 (78.7%)	6 (85.7%)
	Two (e)	11 (16.2%)	10 (16.4%)	1 (14.3)
	Three (e)	3 (4.4%)	3 (4.9%)	0 (0.0%)
Most frequently used antibiotics (b)	Meropenem	21 (24.7%)	18 (23.4%)	3 (37.5%)
	Ceftriaxone	14 (16.5%)	12 (15.6%)	2 (25.0%)
	Piperacillin-Tazobactam	8 (9.4%)	7 (9.1%)	1 (12.5%)
	Ciprofloxacin	8 (9.4%)	8 (10.4%)	0 (0.0%)
	Cefepime	6 (7.1%)	6 (7.8%)	0 (0.0%)
	Augmentin	5 (5.9%)	5 (6.5%)	0 (0.0%)
	Ceftazidime-avibactam	3 (3.5%)	2 (2.6%)	1 (12.5%)
	Trimethoprim-sulfamethoxazole	3 (3.5%)	3 (3.9%)	0 (0.0%)
	Ceftazidime	3 (3.5%)	3 (3.9%)	0 (0.0%)
	Vancomycin	3 (3.5%)	2 (2.6%)	1 (12.5%)
	Other (≤ 2 cases each) (c)	11 (12.9%)	11 (14.3%)	0 (0.0%)
Route of antibiotic administration (d)	Intravenous (IV)	66 (77.6)	58 (75.3%)	8 (100%)
	Oral	19 (22.4)	19 (24.7%)	0 (0.0%)
Duration of antibiotic courses, Median (IQR) days		6.75 (7.0)	7.0 (7.0)	6.0 (8.0)

**Note: (a)** Percentages for the “Number of antibiotic types” are calculated from 68 patients who received antibiotics, including 61 with CDI without recurrence and 7 with R-CDI, **(b)** Percentages for total specific antibiotics are calculated from 85 antibiotic courses recorded across 68 patients, percentages in CDI calculated from 77 antibiotic courses and percentage In R-CDI are calculated from 8 antibiotic courses, **(c)** Includes isolated uses of Clindamycin, Gentamicin, cefuroxime, Cefotaxime cefazolin, amoxicillin, trimethoprim, and doxycycline, **(d)** Routes were available for all 85 antibiotic courses (IV = 66, Oral = 19). **(e)** Of the 68 patients who received antibiotics, 14 (20.6%) received combination therapy involving two or more agents concurrently or sequentially. Percentages of total calculated from 85 antibiotic courses, percentages in CDI calculated from 77 antibiotic courses and percentage in R-CDI are calculated from 8 antibiotic courses, some values may not sum to 100% due to rounding.

**Abbreviation:** IV: Intravenous; IQR: Interquartile range.

### 3.3. Acid suppressant exposure

Acid suppressant exposure was examined for its contributory role in CDI, with stratification by recurrence. Table 3 sequentially details overall exposure, followed by Acid suppressant and route among users to provide targeted insights. Acid suppressants were used in 57.0% (n = 65) of patients, compared with 43.0% (n = 49) with no use or missing data, and usage was more frequent in R-CDI (76.9%). Omeprazole was the primary agent (92.3% of exposures, n = 60), followed by esomeprazole (7.7%, n = 5). Among Acid suppressant users, intravenous administration occurred in 63.1% (n = 41) and oral in 36.9% (n = 24). This widespread exposure, particularly elevated in recurrent cases, identifies acid suppression as a potential modifiable factor influencing CDI recurrence (Table 3).

**Table 3:** Acid-suppressant use, type, and administration route among hospitalized patients with CDI (N = 114).

Characteristic	Category	Total n (%) (n = 114)	CDI without recurrence (n = 101)	Recurrent CDI (R-CDI) (n = 13)
**Acid-suppressant use**	Used	65 (57.0%)	55 (54.5%)	10 (76.9%)
	Did not use/ No data	49 (43.0%)	46 (45.5%)	3 (23.1%)
**Type of acid suppressant** (a)	Omeprazole	60 (92.3%)	51 (92.7%)	9 (90.0%)
	Esomeprazole	5 (7.7%)	4 (7.3%)	1 (10.0%)
**Route of administration** (b)	Intravenous (IV)	41 (63.1%)	34 (61.8%)	7 (70.0%)
	Oral	24 (36.9%)	21 (38.2%)	3 (30.0%)

**Note:** Percentages in the “Total” column are based on the overall study population (N = 114).

Percentages within CDI and R-CDI columns are calculated relative to group totals (101 and 13 patients, respectively), For rows under Type and Route, percentages are calculated from patients who received acid suppression (Total n = 65; CDI n = 55; R-CDI n = 10).

**(a)** Among the 65 patients who received acid suppression, omeprazole was the predominant agent (> 90%).

**(b)** Among acid-suppression users (n = 65), 41 (63.1%) received therapy intravenously and 24 (36.9%) orally.

**Abbreviation:** IV: Intravenous; R-CDI: Recurrent *Clostridioides difficile* infection.

### 3.4. CDI clinical course and treatment

Clinical comorbidities (malignancy, previous surgery), treatment provision, current regimens, and initial treatments in recurrent cases were compared by recurrence status to identify factors influencing CDI management and outcomes. The analysis was organized by comorbidities, treatment status and types, and initial regimens in R-CDI to highlight therapeutic patterns and associations. Malignancy was present in 32.5% (n = 37) of patients, more commonly in R-CDI (53.8%), while prior surgery affected 15.8% (n = 18) overall but none in R-CDI. Treatment was administered to 79.8% (n = 91), with no treatment or missing data in 20.2% (n = 23). Among treated patients, metronidazole was most frequent (64.8%, n = 59), followed by metronidazole plus vancomycin (20.9%, n = 19) and vancomycin monotherapy (12.1%, n = 11); combination therapy was significantly more common in R-CDI (58.3% vs. 15.2%, p = 0.013). In recurrent cases with recorded initial treatment (n = 9), metronidazole was used in 66.7% (n = 6), combination in 22.2% (n = 2), and doxycycline in 11.1% (n = 1). The observed escalation to combination regimens in recurrent episodes reflects adaptive clinical responses (Table 4).

**Table 4:** Clinical and Treatment Characteristics by Recurrence Status among Patients with CDI (N = 114).

Characteristic	Category	Total n (%)	CDI without recurrence (n = 101)	Recurrent CDI (R-CDI) (n = 13)
**Malignancy**	Yes	37 (32.5%)	30 (29.7%)	7 (53.8%)
	No	77 (67.5%)	71 (70.3%)	6 (46.2%)
**Previous surgery**	Yes	18 (15.8%)	18 (17.8%)	0 (0.0%)
	No	96 (84.2%)	83 (82.2%)	13 (100.0%)
**Treatment status**	Treated	91 (79.8%)	79 (78.2%)	12 (92.3%)
	No treatment/ No data	23 (20.2%)	22 (21.8%)	1 (7.7%)
**Current treatment (type)** (a)	Metronidazole	59 (64.8%)	55 (69.6%)	4 (33.3%)
	Metronidazole + Vancomycin	19 (20.9%)	12 (15.2%)	7 (58.3%)
	Vancomycin	11 (12.1%)	10 (12.7%)	1 (8.3%)
	Other	2 (2.2%)	2 (2.5%)	0 (0.0%)
**Initial treatment among patients who later developed recurrence** (b)	Metronidazole	6 (66.7%)	—	6 (66.7%)
	Metronidazole + Vancomycin	2 (22.2%)	—	2 (22.2%)
	Doxycycline	1 (11.1%)	—	1 (11.1%)

**Note:** Percentages in the “Total” column are based on the overall population (N = 114).

Percentages within CDI and R-CDI columns are calculated relative to group totals (101 and 13 patients, respectively). **(a)** Current treatment analysis limited to 91 treated patients (79 with CDI, 12 with R-CDI).

Significant association detected between treatment type and recurrence (Fisher Exact Test = 9.649, *p* = 0.013).

**(b)** Initial treatment among recurrence cases (n = 9 with recorded data) included metronidazole (66.7%), metronidazole + vancomycin (22.2%), and doxycycline (11.1%). Some percentages may not sum to 100% due to rounding.

**Abbreviation:** R-CDI – recurrent *Clostridioides difficile* infection.

### 3.5. Associations and predictive analysis

Bivariate associations and multivariable logistic regression were used to find factors linked to CDI recurrence. We started with simple tests (bivariate) and then adjusted for multiple factors in a regression model. Table 5 shows bivariate results grouped by risk factors. Table 6 lists all factors tested in the regression model. In bivariate tests, antibiotic use (*p* = 0.872) and medical history (*p* = 0.938) showed no link to recurrence. Acid suppressant use did show a link (*p* = 0.041). Malignancy was more common in R-CDI (53.8%) than non-recurrent CDI (29.7%), but not significantly. For the regression model (n = 114 patients), we included factors that were clinically important or had *p* < 0.20 in bivariate tests: age, sex, acid suppressant use, malignancy, and treatment type. We excluded nationality and ward because they had no link. The model fit the data well (Hosmer–Lemeshow test: χ² = 3.90, *p* = 0.866). It explained 37.5% of the variation in recurrence (Nagelkerke R^2^ = 0.375) and correctly classified 84% of cases. Only metronidazole plus vancomycin treatment was significantly linked to higher recurrence risk (odds ratio = 11.29, 95% CI 1.13–112.42, *p* = 0.039). Although not significant, there were trends for higher risk with malignancy (odds ratio = 2.94, 95% CI 0.78–11.12, *p* = 0.112) and acid suppressant use (odds ratio = 1.85, 95% CI 0.39–8.83, *p* = 0.440). Full model results are in Table 6. Combination treatment was the main predictor of recurrence.

**Table 5:** Bivariate Associations Between CDI Recurrence and Risk Factors (N = 114).

Risk Factor	Recurrent CDI (R-CDI) (n = 13)	CDI without recurrence (n = 101)	Total N	% of Total	*p*-value
**Antibiotic Exposure (a)**					0.872
Not Used or No Data	6	40	46	40.4	
Used	7	61	68	59.6	
**Acid-suppressant use (b)**					0.041
Used	10	55	65	57	
Not Used	3	46	49	43	
**Medical History**					0.938
Malignancy	7	30	37	32.5	
Gastrointestinal Diseases	3	20	23	20.2	
No Diagnosis	1	14	15	13.2	
Cardiovascular Diseases	1	11	12	10.5	
Immune System Disorder	0	4	4	3.5	
Miscellaneous Conditions	0	7	7	6.1	
Infectious Diseases	0	6	6	5.3	
Endocrine and Metabolic Disorders	1	3	4	3.5	
Renal and Urological Disorders	0	4	4	3.5	
Neurological Disorders	0	2	2	1.8	
**Total**	13	101	114	100.0	

**Note: (a)** Detailed antibiotic class distribution and administration routes are presented in Table 2.

**(b)** Acid-suppressant characteristics (agent type and route) are described in Table 3.

Statistical significance was assessed using Fisher’s exact test; *p* < 0.05 was considered significant.

Some values may not sum to 100% due to rounding.

**Abbreviations:** R-CDI: Recurrent *Clostridioides difficile* infection.

**Table 6:** Multivariable Logistic Regression for Predictors of CDI Recurrence (N = 114).

Predictor	B	S.E.	Wald	*p*-value	Odds Ratio (OR)	95% CI for OR
Age (years)	0.011	0.013	0.732	0.392	1.01	0.99–1.04
Sex (Female vs. Male)	0.381	0.682	0.312	0.576	1.46	0.39–5.57
Acid suppressant use (Yes vs. No)	0.616	0.797	0.597	0.440	1.85	0.39–8.83
Malignancy (Yes vs. No)	1.078	0.679	2.520	0.112	2.94	0.78–11.12
Metronidazole (vs. all others)	0.423	1.206	0.123	0.725	1.53	0.14–16.22
Metronidazole + Vancomycin (vs. all others)	**2.424**	**1.173**	**4.272**	**0.039**	**11.29**	**1.13–112.42**
Vancomycin alone (vs. all others)	1.016	1.512	0.451	0.502	2.76	0.14–53.45
Constant	−4.588	1.363	11.327	0.001	—	—

**Note.** Model summary: −2 Log Likelihood = 31.45; Cox & Snell R^2^² = 0.182; Nagelkerke R² = 0.375; Hosmer–Lemeshow χ² = 3.90, *p* = 0.866; classification accuracy = 84%.

Logistic regression was performed using data from all eligible patients (N = 114).

Predictors entered were age, sex, malignancy, acid-suppressant use, and antibiotic regimen (metronidazole, vancomycin, or combination therapy). Variables were selected based on clinical relevance and bivariate association (*p* < 0.20). Nationality and ward of admission were examined in bivariate analysis but were not significant and thus excluded from the final model.

**Abbreviations:** OR: Odds ratio; CI: confidence interval; SE: standard error; CDI: *Clostridioides difficile* infection; R-CDI: recurrent CDI.

## 4. Discussion

This study provides a comprehensive assessment of the prevalence, risk factors, and outcomes of CDI among hospitalized patients in KAUH a tertiary teaching hospital in Saudi Arabia. The observed prevalence of 10.8% is higher than rates reported in previous Saudi studies, including 9.1% at KAUH and 8.8% among inflammatory bowel disease patients at the same institution [23,24], and surpasses the 6.8% reported in the western region of Saudi Arabia [25]. Nationally, CDI prevalence has ranged between 4.6% and 23.6% across the Arabian Peninsula over the past two decades [26], while incidence at a Riyadh tertiary facility was lower at 3.5 per 10,000 patient days [21]. The relatively high prevalence in our center likely reflects multiple factors, including broad-spectrum antibiotic use, prolonged hospital stays, and increasing comorbidities [21]. Additionally, the westernization of lifestyle implies adoption of high-fat diets, reduced physical activity, and urbanization-linked microbiome changes, and smoking, which are linked to rising Crohn’s disease rates, and may indirectly impact CDI prevalence [27,28]. Though CDI rates remain lower than those in many high-income countries [21], this study emphasizes the growing burden of CDI in Saudi hospitals and highlights the need for robust infection prevention and antibiotic stewardship strategies.

Our cohort demonstrated a broad age range and balanced gender distribution, aligning with studies suggesting that observed gender differences in CDI incidence are largely driven by antibiotic prescribing practices rather than biological factors [29–32]. The higher proportion of non-Saudi patients with CDI may indicate variation in healthcare access, antibiotic use, or comorbid conditions among different nationalities [33]. Notably, CDI prevalence was highest in emergency and pediatric wards, but this pattern likely reflects hospital-acquired CDI (HA-CDI) rather than community-associated CDI (CA-CDI). Saudi studies consistently report that 70–73% of CDI cases are hospital-onset [21,22]. The frequent hospitalization and immunosuppression observed in pediatric oncology and chronic disease populations likely contributed to this distribution, underscoring the importance of careful ward-level surveillance and targeted infection control [34,35].

In the present study, antibiotics were administered to 59.6% of patients prior to CDI diagnosis, confirming that antibiotic exposure remains the strongest modifiable risk factor for initial CDI acquisition. The most frequently prescribed agents were meropenem (24.7% of courses) and ceftriaxone (16.5% of courses), consistent with the predominant use of broad-spectrum intravenous antibiotics observed in previous Saudi reports from Riyadh and the western region [21–23]. This pattern reflects the common clinical practice of empiric broad-spectrum coverage in hospitalized and critically ill patients in our setting. Carbapenems, third-generation cephalosporins, and fluoroquinolones are well-recognized drivers of CDI due to their profound effects on gut microbiota [36,37]. The high prevalence of meropenem use underscores the urgency of strengthening stewardship programs to curb unnecessary broad-spectrum antibiotic use and limit CDI risk.

In the present study, acid suppressants were used in 57.0% of CDI patients, with higher exposure in those who developed recurrence (76.9% vs. 54.5%; *p* = 0.041 on bivariate analysis). Omeprazole was by far the most frequently prescribed agent (92.3% of all acid-suppressant exposures). These findings confirm the high prevalence of proton pump inhibitor (PPI) use in hospitalized CDI patients in our setting. PPIs disrupt gastric acidity and alter gut microbiota, facilitating *C. difficile* colonization [38]. Large-scale studies have consistently shown that PPI use increases the risk of initial CDI more than histamine-2 receptor antagonists, with reported risk elevations of 38–74% [39–41]. Some evidence also links ongoing PPI exposure to greater severity and recurrence of CDI, although confounding by comorbidity and indication bias has been noted [42, 43]. In our cohort, the significant bivariate association between acid-suppressant use and recurrence attenuated after multivariable adjustment (aOR 1.85, 95% CI 0.39–8.83, p = 0.440), likely reflecting the small number of recurrent events and overlapping risk factors such as advanced age and malignancy. Nevertheless, the high prevalence of PPI use, particularly omeprazole, reinforces the need for judicious prescribing and regular review of indications in hospitalized patients at risk of CDI.

Patients with malignancies and other comorbidities are well-documented as being at high risk for CDI and its complications [44–46]. In our study, malignancy was present in 32.5% of patients, lower than the 46% reported by Althaqafi et al. [19] at the same institution. This discrepancy may reflect differences in patient populations or study periods but nonetheless highlights the importance of targeted surveillance in oncology patients, particularly those with hematological malignancies. Frequent chemotherapy, antibiotic prophylaxis, and prolonged hospitalizations likely contribute to CDI susceptibility and recurrence in this group [47,48]. In our cohort, 15.8% of CDI patients had a history of surgery, though we did not stratify by procedure type. Literature shows that surgical interventions, particularly gastrointestinal procedures, can disrupt gut microbiota and mucosal barriers, increasing susceptibility to CDI and severe outcomes [49,50]. Post-surgical patients are also at risk for prolonged hospitalizations, complications, and higher recurrence rates [50–52]. While surgery was not a significant predictor of recurrence in our analysis, its presence among CDI patients highlights a subgroup with elevated clinical complexity and warrants closer monitoring.

CDI recurrence was observed in 11.4% of our cohort, consistent with global estimates of 10–25% recurrence following an initial episode [51,53–55]. The risk of further recurrences increases substantially, reaching 40–65% after one or two prior episodes [51]. Multivariable logistic regression explained 37.5% of the variance in recurrence (Nagelkerke R^2^ = 0.375). Only metronidazole plus vancomycin combination therapy was independently associated with higher recurrence risk (OR = 11.29, 95% CI 1.13–112.42, *p* = 0.039). Malignancy (OR = 2.94, 95% CI 0.78–11.12, *p* = 0.112) and acid suppressant exposure (OR = 1.85, 95% CI 0.39–8.83, p = 0.440) showed non-significant trends toward increased risk, likely limited by the small number of recurrent events (n = 13) and resulting wide confidence intervals. Literature consistently identifies malignancy as a key recurrence risk factor, with odds ratios of 1.5–1.8 in large population-based studies and cancer-specific cohorts, findings echoed in the recent multicenter CIRCA study [54,56,57].

Although prior studies consistently report a dose-dependent relationship between antibiotic exposure and CDI recurrence, with recurrence risk rising alongside both the number of antibiotic classes and duration of use [58–60], our analysis did not identify such an association. In our cohort, recurrence occurred at comparable rates across patients with one, two, or three antibiotic exposures, and no statistically significant trend was observed (*p* = 0.872). This divergence may reflect sample size limitations or confounding factors such as underlying comorbidities, which could have masked the expected relationship. These findings emphasize that recurrence in our cohort reflects established global patterns and highlight the importance of ongoing surveillance and targeted prevention strategies especially in oncology patients, elderly individuals, and those with multiple comorbidities to mitigate morbidity and mortality associated with recurrent CDI.

Treatment practices in our study were dominated by metronidazole (64.8%), with vancomycin or combination therapy reserved for severe or recurrent cases. This pattern reflects historical practice norms and formulary considerations in Saudi Arabia, where no national CDI-specific guidelines exist; instead, clinicians often follow international standards like IDSA/SHEA while adapting to local resources. A 2023 study in Jeddah found comparable outcomes between metronidazole and vancomycin for non-fulminant CDI, noting metronidazole’s continued use as a reasonable option despite the 2017 IDSA/SHEA shift away from it as first-line for non-severe cases [61]. However, extensive evidence now shows that metronidazole is less effective than vancomycin or fidaxomicin in achieving sustained cure, particularly in high-risk patients [62–64]. Systematic reviews report recurrence rates of 20–28% following metronidazole, with some studies showing rates as high as 27.1% compared to 24.0% for vancomycin [63,64]. In our cohort, 66.7% of recurrent cases had initially received metronidazole, yet the overall recurrence rate among metronidazole-treated patients did not significantly differ from other regimens, likely reflecting the small sample size rather than equivalence in efficacy. These findings reinforce updated guidelines from IDSA/SHEA and the American College of Gastroenterology, which recommend vancomycin or fidaxomicin as preferred initial treatments and bezlotoxumab for recurrence prevention [17,50,65]. A 2020 analysis at a Saudi tertiary center showed that compliance with IDSA/SHEA and ACG guidelines improved clinical cure (76.5%) and reduced mortality, underscoring the value of guideline adherence in our context [66]. Future initiatives should focus on expanding access to fidaxomicin and adjunctive therapies while reinforcing clinician education. Framing this evolution within a global context underscores that these changes represent advancements in CDI management strategies rather than criticism of local policies.

## 5. Limitations

Several limitations must be acknowledged. Some CDI cases were diagnosed solely based on NAAT testing for *C. difficile* toxin genes, without supplementary enzyme immunoassay screening for glutamate dehydrogenase antigen or toxins A and B. This could impact diagnostic accuracy, as NAAT alone cannot differentiate between CDI colonization and true infection. Additionally, the retrospective design of the study may introduce selection bias, and reliance on hospital records and self-reported data for antibiotic and acid suppressant use may affect accuracy. The study’s single-center nature may also limit the generalizability of the findings. Future multi-center and prospective studies are needed to provide a broader understanding of CDI prevalence and management.

## 6. Conclusion

This study documents a CDI prevalence of 10.8% among hospitalized patients at a Saudi teaching tertiary center, with recurrence occurring in 11.4% of cases. Acid-suppressant exposure was significantly associated with recurrence on bivariate analysis (*p* = 0.041) and metronidazole plus vancomycin combination therapy emerged as the only independent predictor of recurrence (OR 11.29, 95% CI 1.13–112.42, *p* = 0.039). Despite current international guidelines favoring vancomycin or fidaxomicin as first-line therapy, metronidazole monotherapy remained the dominant treatment (64.8%). These findings highlight the persistent reliance on older regimens, the potential role of acid suppression in recurrence, and the heightened risk in patients with malignancy and advanced age. Strengthening antibiotic and acid-suppressant stewardship, improving access to guideline-recommended therapies, and implementing targeted surveillance in high-risk wards (oncology, pediatrics, emergency) are essential to reduce the burden of both initial and recurrent CDI in Saudi Arabian hospitals.
